# Following a natural experiment of guideline adaptation and early implementation: a mixed-methods study of facilitation

**DOI:** 10.1186/1748-5908-7-9

**Published:** 2012-02-06

**Authors:** Elizabeth J Dogherty, Margaret B Harrison, Cynthia Baker, Ian D Graham

**Affiliations:** 1 School of Nursing, Queen's University, Rm 200 - 78 Barrie St., Kingston, ON K7L 3N6 Canada; 2 Canadian Association of Schools of Nursing, 99 Fifth Ave., Suite 15, Ottawa, ON K1S 5K4 Canada; 3 Canadian Institutes of Health Research, 160 Elgin St., 9th Floor AL 4809A, Ottawa, ON K1A 0W9 Canada

**Keywords:** facilitation, facilitator, evidence-based practice, nursing, guideline

## Abstract

**Background:**

Facilitation is emerging as an important strategy in the uptake of evidence. However, it is not entirely clear from a practical perspective how facilitation occurs to help move research evidence into nursing practice. The Canadian Partnership Against Cancer, also known as the 'Partnership,' is a Pan-Canadian initiative supporting knowledge translation activity for improved care through guideline use. In this case-series study, five self-identified groups volunteered to use a systematic methodology to adapt existing clinical practice guidelines for Canadian use. With 'Partnership' support, local and external facilitators provided assistance for groups to begin the process by adapting the guidelines and planning for implementation.

**Methods:**

To gain a more comprehensive understanding of the nature of facilitation, we conducted a mixed-methods study. Specifically, we examined the role and skills of individuals actively engaged in facilitation as well as the actual facilitation activities occurring within the 'Partnership.' The study was driven by and builds upon a focused literature review published in 2010 that examined facilitation as a role and process in achieving evidence-based practice in nursing. An audit tool outlining 46 discrete facilitation activities based on results of this review was used to examine the facilitation noted in the documents (emails, meeting minutes, field notes) of three nursing-related cases participating in the 'Partnership' case-series study. To further examine the concept, six facilitators were interviewed about their practical experiences. The case-audit data were analyzed through a simple content analysis and triangulated with participant responses from the focus group interview to understand what occurred as these cases undertook guideline adaptation.

**Results:**

The analysis of the three cases revealed that almost all of the 46 discrete, practical facilitation activities from the literature were evidenced. Additionally, case documents exposed five other facilitation-related activities, and a combination of external and local facilitation was apparent. Individuals who were involved in the case or group adapting the guideline(s) also performed facilitation activities, both formally and informally, in conjunction with or in addition to appointed external and local facilitators.

**Conclusions:**

Facilitation of evidence-based practice is a multifaceted process and a team effort. Communication and relationship-building are key components. The practical aspects of facilitation explicated in this study validate what has been previously noted in the literature and expand what is known about facilitation process and activity.

## Background

Integration of evidence into practice remains a poorly understood, complex process. Consequently, research into changing practice to reflect best available evidence has become an important health services area of enquiry. There is increasing interest in the design and evaluation of ways to enhance research utilization. Facilitation is emerging as a method for encouraging evidence uptake in clinical practice across healthcare disciplines and particularly in nursing. Early work in this area was led by Kitson *et al. *[1] who developed a framework indicating that successful implementation is dependant on the relationship between three key factors: the nature of the evidence, the quality of the context, and facilitation. Facilitators play an important role in assisting individuals and teams with identifying what needs to change and how to make these changes to integrate evidence into practice [2].

Harvey *et al. *[3] conducted a concept analysis of facilitation to further develop the framework and examined a range of healthcare literature published from 1985 to 1998. They discovered that facilitation does involve assisting others to make changes in practice with the purpose 'ranging from a discrete task-focused activity to a more holistic process of enabling individuals, teams and organizations to change' [3]. However, descriptions and rigorous evaluations of facilitation were lacking. They concluded that the concept is only partially developed and further research is required to understand how it relates to evidence uptake.

To determine how the concept of facilitation has evolved over the last decade, particularly in light of rapid advancements in the field of implementation, we conducted a focused literature review [4]. Particular attention was given to the practical elements of the concept and what is entailed to put facilitation into action. Several things emerged from this review. Facilitation continues to involve supporting and enabling practitioners to change practice through evidence uptake. There is an emerging focus on relationship-building and communication as part of the role [5,6]. In line with Harvey *et al*.'s [3] findings, we found that facilitation ranges from providing task-oriented assistance to enabling individuals and groups to alter their ways of thinking and working.

We uncovered new components and themes in conceptualizations of facilitation in knowledge uptake, specifically: facilitation is an individual role as well as a process (*i.e*., it does not always have to be a 'facilitator' assuming the role-groups may engage in facilitation processes); project management and leadership are emerging as aspects of facilitation with facilitators taking on project leadership roles; tailoring facilitation to the local context is critical; and a growing focus on evaluation and linking outcomes with action (*i.e*., observation of positive outcomes resulting from changing practice) [4].

Several authors indicate that there is some degree of overlap between facilitation and a number of other change agent roles [3,6]. For example, Harvey *et al. *[3] found that facilitation models contain elements of educational outreach approaches, sometimes referred to as academic detailing. The distinction between the various roles (*i.e*., facilitators, opinion leaders, champions, linking agents, and educational outreach workers) may lie in whether the change agent is internal or external to the environment where change is to take place in addition to the underlying theoretical perspective from which the agent functions [3,6]. Opinion leaders are typically internal to the organization and educational outreach workers tend to be external whereas facilitators may be internal or external [3]. In terms of theoretical orientation, facilitators use group dynamics and skills to promote change while opinion leaders and other change agents rely on their level of expertise and knowledge [6]. Others suggest that facilitation is more general than other change agent roles and facilitators likely integrate other implementation strategies while facilitating such as providing education and using audit and feedback data [5]. There continues to be a lack of conceptual clarity.

In general, we found in the more recent literature that the concept is emerging as a more practical and applied process, but further research is needed into how facilitation is being used to make changes in nursing practice. Facilitation could be considered a distinct intervention; however, there is a need for studies defining and specifically examining facilitation to understand its contribution to successful implementation across various types of projects and contexts [5]. To operationalize facilitation, a clear and more comprehensive understanding of what facilitators are doing in real situations to enable changes in nursing practice is a needed next step. Rather than proactively designing a specific facilitation intervention, we may gain more understanding if we follow groups and track the way they facilitate a journey to evidence-based practice (EBP). This would lay the groundwork for the design and delivery of practical strategies for EBP where facilitation is a key component.

### The Partnership study

We conducted this research alongside a larger, natural experiment case-series study. The Canadian Partnership Against Cancer, also known as the 'Partnership,' is a Pan-Canadian initiative involved in knowledge translation activity for improved care through guideline use [7]. In this case-series study, five self-identified cases volunteered to use a systematic methodology [8] to adapt existing clinical practice guidelines for Canadian use. The focus of study was groups beginning evidence implementation through adaptation of a guideline and their use of the prescribed methodology in the adaptation work. An in-depth process evaluation followed their course in selecting, adapting, and planning for implementation of these guidelines. Detailed accounts of this process were documented for each case.

Each of the five cases had a dedicated local facilitator and two external facilitators were also available as needed. Local facilitators were 'in the field' actively working with cases (*i.e*., embedded in the setting both geographically and socially) while external facilitators were university-based and off site (*i.e*., external to the setting) providing strategic and methodological support. Each case had an appointed lead and/or co-leads for their respective projects, and these individuals could be considered content experts in their field. Three of the five cases focused on nursing care and became the subject matter of this facilitation enquiry.

### Study conceptualization

**Figure 1:**
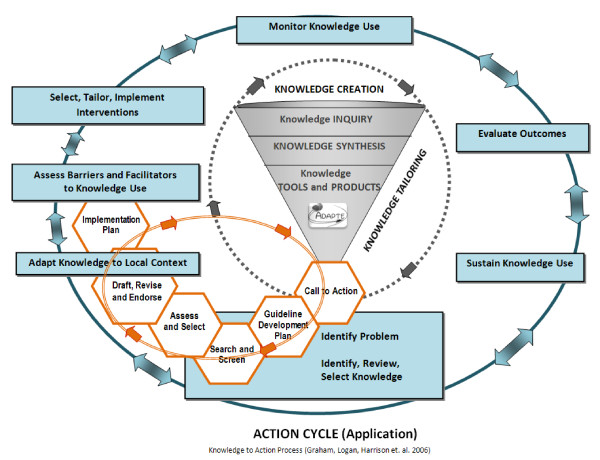
**Knowledge to action process with guideline adaptation integrated**. Harrison, M. B., & van den Hoek, J. for the Canadian Guideline Adaptation Study Group. CAN-IMPLEMENT^©^: Guideline Adaptation and Implementation Planning Resource. Queen's University School of Nursing and Canadian Partnership Against Cancer, Kingston, Ontario, Canada, April 2010 Version 1.0. PG. 2; http://www.cancerview.ca/portal/server.pt/community/initiatives_and_resources/473/cancer_guideline_adaptation_and_implementation_project MODIFIED from original KTA process [9]: permission obtained to use the figure from Dr. Curtis Olson, Editor-in-Chief, *The Journal of Continuing Education in the Health Professions*

Broadly, the 'Partnership' initiative is driven by the Canadian Institutes of Health Research knowledge to action (KTA) process [9,10]. The process includes both a knowledge creation and an application cycle. The six critical work elements in guideline adaptation fit within the broader KTA process (Figure 1).

Based on findings of our literature review, we conceptualized facilitation as a multifaceted process encompassing a number of different activities [4]. This study was driven by and builds upon the review by investigating if facilitation in practice reflects facilitation as it is described in the literature. We used the results of the review to develop a synoptic table outlining the major elements of facilitation of EBP in nursing [4]. The table outlines four stages of facilitation, namely: planning for change, leading and managing change, monitoring progress and ongoing implementation, and evaluating change. Within each of these stages are 11 groupings of activity with 46 specified activities and actions related to facilitation. For example, in the planning for change stage, there are two groupings of activity: increasing awareness and developing a plan. Specific activities associated with increasing awareness are: highlighting a need for practice change and selecting an area for change relevant to staff/recognized as a priority. The review findings provided a preliminary taxonomy of the practical aspects of facilitation and the associated activities in moving evidence into nursing practice.

### Purpose and objectives

The aim of this study was to gain a comprehensive understanding of the activities and skills of individuals actively engaged in facilitation and to describe the process of facilitation occurring within the 'Partnership' case-series study. The cases represented various areas of cancer care and guideline topics for use in nursing practice. The following questions guided the enquiry:

1. What activities are performed by appointed facilitators (local and external) in the 'Partnership' study?

2. How do activities undertaken by these facilitators relate to evidence and theory in the literature?

3. Are there facilitation activities performed by those other than the appointed facilitators?

4. What are facilitators' perceptions of the most important elements of facilitation based on their practical experiences?

5. What do facilitators identify as the skills and knowledge required for effective facilitation?

## Methods

We utilized a mixed-methods design employing case audit and a focus group interview. These methods allowed us to apply another lens for an in-depth investigation of facilitation from the detailed overall process tracking undertaken by the 'Partnership' study research team and a focus group interview with facilitators as the process was occurring. We were able to link the two forms of data (quantitative and qualitative) sequentially by having the focus group build on the case audit findings [11].

### Participants and sites

Three nursing cases from the 'Partnership' study representing a range of experience and contexts formed the facilitation study set. These cases represented different areas of nursing practice and levels of guideline implementation (*i.e*., local, regional) and possessed variation in clinical guideline focus (assessment and management recommendations) and the scope of implementation from a single setting to multiple settings. All had a goal to improve a certain aspect of cancer care (Table 1).

**Table 1:** Partnership study case descriptions

Case:	1: Nurse Tele Practice	2: Breast Cancer Wound Care	3: Distress Management
**Scope of Implementation**	Pan-Canadian	Local	Regional

**Clinical Practice Guideline Focus**	Point-of-care, supportive symptom management for patients receiving chemotherapy, radiation, or palliative care	Best practices for local wound care in breast cancer patients following radiotherapy	Distress management in adult cancer patients across the continuum of care

**Target Guideline Users**	Front-line telephone triage nurses	Front-line nurses/radiotherapy technicians	Front-line care providers

The other two cases involved in the larger case-series study were excluded. One case, comprised primarily of physicians and pharmacists, was excluded as their main focus was on developing guidelines *de novo *as opposed to adapting existing guidelines. The fifth case was excluded as the group was adapting a guideline regarding the assessment and management of distress in cancer patients, similar to case three outlined in Table 1, however with a national scope of implementation. We chose to investigate the case adapting a similar guideline at the regional level to provide diversity in the sample in regards to scope of implementation of the cases and avoid redundancy in terms of guideline focus/topic area.

Selected facilitators (four local, two external) involved with the cases were invited and consented to participate in the focus group interview. Participants were actively involved in explicit facilitative activities and supported cases through the guideline adaptation and implementation process. These individuals came from various backgrounds including project management, research and data management, teaching, and nursing. All were female. Facilitation was defined and operationalized case by case locally and was not prescribed. If the group required assistance, they asked and it was tracked as it naturally occurred.

### Data collection

We undertook two stages of data collection: case audit and focus group interview. Case-audit data were obtained from the individual case manuals for the three nursing cases. The manuals were comprehensive data sets of the process tracking containing: contextual information; case documents; email correspondence between case members (participants involved with the guideline work); meeting minutes/field notes; a case log (indicating date, activity, who was involved, and a brief summary); a case liaison (detailing external facilitator contact with the case); and a process timeline which mapped progress through the guideline adaptation phases outlined in Figure 1. The cases joined the larger case-series study at different points in time therefore, the data collection period for each case audit varied. We examined 16 months of documentary data for case one, 11 months for case two, and 17 months for case three. Additionally, each case was different in terms of their progress through the adaptation phases and the process was iterative and not linear. Some steps required work over longer periods of times and several cycles (*i.e*., searching and screening the literature). In general however, the cases took between 12 and 24 months to agree on the guideline recommendations and draft the initial guideline.

The facilitation audit was undertaken with the audit tool formulated from the emerging taxonomy based on the literature review [4]. The tool consisted of the four major stages of facilitation: planning for change, leading and managing change, monitoring progress and ongoing implementation, and evaluating change. The 11 groupings of activity with 46 specified activities and actions related to facilitation were evaluated against the data extracted from the case audit (Additional file 1: Facilitation case-audit tool). The audit tool was reviewed by a knowledge translation panel of researchers and experienced facilitators for feedback on its validity and feasibility of administration.

To avoid predetermining results based on the literature, we employed a multi-step approach to data collection. First, one author (EJD) examined all of the documentation for each individual case to determine activities performed by appointed facilitators and created an exhaustive list with corresponding examples per case. The general definition of facilitation put forth by Stetler *et al. *[5] broadly guided what was considered facilitation activity: 'a deliberate and valued process of interactive problem solving and support that occurs in the context of a recognized need for improvement and a supportive interpersonal relationship' [5]. These results were reviewed and discussed with the investigator team.

Next, EJD examined the case data a second time using the theoretically-driven audit tool searching for examples of the facilitation activities previously noted in the literature. Activities that did not fit into these predetermined categories were kept separate for later categorization. These results were also reviewed and discussed with the other investigators.

Finally, the list of activities and examples from the first run through of the data were examined to determine their fit with the audit tool and categorized accordingly. EJD checked to see if where these activities were inserted in the grid matched with where they were categorized during the second round of data collection using the audit tool. Differences were noted. Again, activities that did not fit were kept separate for later categorization. Agreement on these was confirmed and discussed with the investigator team.

Following the audit, the data manager of the Partnership study who was familiar with all three cases checked the data sets to confirm that examples of facilitation activities supported the different categories of facilitation as outlined in the audit tool. Subsequently, EJD and the data manager met to challenge interpretations and discuss any disagreements in order to reclassify the activities where necessary and to decide where the newly identified actions fit into the overall synoptic table [4]. Detailed notes were kept of decisions made and changes were tracked.

Three complete facilitation data sets resulted, one for each case. Again, the case data were examined in an open-ended manner first in an attempt to avoid forcing the placement of evidence or activities found into the predetermined categories outlined in the audit tool. The intent was to capture facilitation activity regardless of who performed it. Thus, we noted whether the activities identified were performed by individuals other than the assigned facilitators.

Following completion of the audit, EJD conducted a 50-minute focus group interview with facilitators (n = 6). The intent was to gather their perspectives on the following three key areas: the nature of facilitation and the most important elements; the key elements of facilitation identified in the literature review and case audit and whether they reflected their experiences and if there was anything misrepresented or missing; and the skills and knowledge required for effective facilitation.

We used the focus group interview as a means of confirming and modifying the characteristics of facilitation identified in the literature review and case audit. The session was audio-recorded and transcribed.

### Data analysis

We analyzed the case-audit data using a content analysis approach to determine if each facilitation activity presented itself in the case documentation, by whom it was performed, and the recipient of the action. Additional information and examples of activities were formulated into new or existing categories. This information was synthesized then compared across cases to gain an overall description of facilitation and identify any unique, additional elements.

For each case, EJD formulated categories of facilitation into a table to determine whether facilitators or case members performed facilitation activities. This information was sorted into the type of facilitation, which was colour-coded based on who was performing the facilitation action (*i.e*., green = local facilitator, red = external facilitator) (Additional file 2: Facilitation across cases). Activities that did not appear in the audit data were explored in the facilitator focus group interview. Interview data were analyzed according to the predetermined topic areas to identify similarities and differences in facilitators' perceptions and experiences.

### Ethical approval

Ethical approval was received from Queen's University Health Sciences and Affiliated Teaching Hospitals Research Ethics Board.

## Results

This study examined the facilitation activity occurring in three cases adapting guidelines for use by nurses and planning for implementation. Each case had a different clinical focus and scope and level of implementation (Table 1). Results are structured and presented in relation to the research questions. It is important to note some general differences between cases. All exhibited varying levels of facilitation activity with particularly intensive facilitation provided by local facilitators. However, cases two and three were more similar than case one. Case one had substantially more direct external facilitation support and more facilitation activities being performed by members of the case themselves. In all cases, but particularly case one, members performed facilitation activities in conjunction with or in addition to the local and external facilitators.

### Activities performed by appointed facilitators

The case documents revealed that appointed facilitators performed an extensive range and number of facilitation activities (Table 2). In summary, four major stages of facilitation were identified (planning for change, leading and managing change, monitoring progress and ongoing implementation, and evaluating change) and within those, 11 groupings of activity with 51 activities noted overall. We found evidence of function related to local and/or external facilitation for almost all of these activities in at least one case but more often in multiple cases. However, there were a few exceptions as noted in Table 2. Activities performed by both local and external facilitators in all of the cases are listed in Table 3.

**Table 2:** Facilitation activities performed by appointed facilitators

Planning for change	
*Increasing awareness *	*Developing a plan*
1) Highlighting a need for practice change 2) Selecting an area for change relevant to staff/recognized as a priority 3) Stimulating critical inquiry and assisting groups to develop/refine specific clinical practice questions 4) Assisting with/performing a formal/informal practice audit *5) Interpreting baseline data and providing feedback/insight into performance gaps 6) Emphasizing enhanced patient outcomes as opposed to poor practice as reason for change	7) Goal-setting and assisting with development of an action plan 8) Helping identify and determine solutions to address potential barriers to EBP 9) Displaying and generating enthusiasm at the start of the project 10) Thinking ahead in the process

**Leading and managing change**	

*Knowledge and data management*	*Project management *
11) Knowledge translation/dissemination (assisting with conducting literature searches, obtaining articles, appraising and summarizing the evidence) 12) Helping to interpret the research and apply it in practice 13) Providing resources/tools for change	14) Identifying a leader 15) Establishing and allocating roles/delegating responsibilities 16) Advocating for resources and change
*Recognizing the importance of context*	*Fostering team-building/group dynamics*
*17) Creating an open, supportive, and trusting environment conducive to change 18) Helping to build in the structures/processes to support staff and help them overcome obstacles 19) Creating local ownership of change 20) Assisting with adapting evidence to the local context 21) Boundary-spanning (addressing organizational systems/culture), managing the different requirements of each discipline/role 22) Tailoring/adapting facilitation services to the local setting	23) Relationship-building 24) Encouraging effective teamwork 25) Enabling individual and group development 26) Encouraging/ensuring adequate participation 27) Increasing awareness of and helping overcome resistance to change 28) Consensus-building (shared decision-making)
*Administrative and project-specific support*	
29) Organizing/scheduling meetings 30) Leading/participating in meetings 31) Gathering information and assembling/distributing reports and materials	32) General planning 33) Providing skills training 34) Taking on specific tasks

**Monitoring progress and ongoing implementation**	

*Problem-solving*	*Providing support*
35) Problem-solving and addressing specific issues 36) Making changes to the developed plan as necessary 37) Networking *Effective communication* 46) Providing regular communication (emails, phone calls) 47) Keeping group members informed 48) Acting as a liaison	38) Mentoring and role-modelling EBP 39) Maintaining momentum and enthusiasm 40) Acknowledging ideas and efforts 41) Providing ongoing support/reassurance and constructive feedback 42) Empowering group members 43) Providing advice/guidance/assistance 44) Being available as needed 45) Ensuring group remains on task and things are not missed (process/methodology is followed)

**Evaluating change**	

*Assessment*	
49) Performing/assisting with evaluation *50) Linking evidence implementation to patient outcomes *51) Acknowledging success, recognizing and celebrating achievements	

*Activities not noted to be performed by recognized facilitators in the case-series data.

**Table 3:** Activities performed by both local and external facilitators across all three cases

Providing resources/tools for change
Tailoring/adapting facilitation services to the local setting
Consensus-building (shared decision-making)
Organizing/scheduling meetings
Leading/participating in meetings
Problem-solving and addressing specific issues
Providing ongoing support/reassurance and constructive feedback
Ensuring group remains on task and things are not missed (process/methodology is followed)
Providing regular communication (emails, phone calls)
Keeping group members informed

Across cases, certain activities tended to cluster around local and/or external facilitators. For example, local facilitators performed three of three of the activities under 'effective communication' in all cases, in addition to four of six under 'administrative and project-specific support,' and two of three under both 'knowledge and data management' and 'problem-solving.' External facilitators performed six of eight of the activities under 'providing support' in all of the cases and two of three under 'effective communication.' In mapping who was assisting whom, local facilitators generally assisted members involved in each of the cases. Aside from the dissimilar case, external facilitators generally provided support and assistance to local facilitators who in turn assisted the case members.

### Congruence with facilitation as described in the literature

We used results of the literature review [4] to develop the initial synoptic table of facilitation activities that was formulated into the audit tool. As such, this method of data collection called for a comparison with the literature in data analysis. In analyzing the audit data, the literature synopsis encompassed most of the activities of appointed facilitators. Four facilitation elements in the literature were not found to be performed by facilitators in these cases. We also identified five additional aspects of facilitation as part of the appointed facilitator role (Table 4). The new activities were primarily associated with the supportive aspect of the role. In particular, being available as needed to the case and ensuring that they remained on task. All of the larger 11 groupings of activity and corresponding four stages of facilitation articulated in the foundational synopsis were able to capture both the existing and newly identified activities of facilitation.

**Table 4:** Missing and new elements of facilitation in relation to facilitation as described in the literature

Activities for which no documented evidence was found in the case-audit data of being performed by recognized facilitators	New activities identified as performed by recognized facilitators
Interpreting baseline data and providing feedback/insight into performance gaps Creating an open, supportive, and trusting environment conducive to change Linking evidence implementation to patient outcomes Acknowledging success, recognizing and celebrating achievements	Displaying and generating enthusiasm at the start of the project Thinking ahead in the process Taking on specific tasks Being available as needed Ensuring group remains on task and things are not missed

### Facilitation-a process beyond an assigned role

Case members performed certain facilitation activities in conjunction with or in addition to the local and external facilitators. For example, members of all three cases had a substantial role in identifying a leader for their projects. Other activities performed by case members as opposed to facilitators in the majority of cases included: highlighting a need for practice change; selecting an area relevant to staff/recognized as a priority; performing a practice audit; and helping to interpret the research and applying it in practice.

Conversely, there were certain areas where substantial facilitation from both external and local facilitators was required and where none of the facilitation activities were performed by case members themselves. These areas included: providing resources/tools for change; tailoring/adapting facilitation services to the local setting; consensus building; problem-solving; and providing ongoing support/reassurance.

### Key elements of facilitation as perceived by facilitators

When facilitators were asked about their perceptions of the most important elements of facilitation based on their practical experiences, some interesting findings emerged. In reviewing the synopsis of facilitation activities discovered in the case audit (Table 2), participants were readily able to relate to these activities and felt that they fit with their experiences. As one local facilitator stated: '...all these tasks listed here I could, I can relate to like oh yeah, I do that, I do that, oh yeah.' Participants also commented that the layout of the activities and stages of facilitation made sense.

Of the 11 major groupings of activities, participants perceived three to be central tenets of facilitation: knowledge and data management; project management; and administrative and project-specific support. Facilitators felt the administrative element was especially important because individuals involved in this work have numerous other responsibilities and priorities. As a result, '...the project lead and the chair don't have time to try and find proper times for teleconferences and to share information.' Participants perceived the administrative and follow-up support as a key factor in driving the work forward. Several specific activities also emerged as central to the role (Table 5).

**Table 5:** Key facilitation activities identified by facilitators

Emphasizing enhanced patient outcomes (as opposed to poor practice as reason for change)	
Identifying a 'qualified' leader	'...you've got to have the right person with the right knowledge'; '...you really have to pick the right leader otherwise you could spend a lot of time and develop a guideline that just wouldn't be all that useful'; someone who has 'charisma' and 'street credibility.'

Increasing awareness of and helping overcome resistance to change	'They don't want to go there because maybe they're afraid a bit about guidelines. And what they really are and you know, who's going to be using them and who's going to be writing them, et cetera...by trying to be positive and supportive and inclusive, gee people are buying in and saying 'this is fantastic, this is great.'

Gathering information and assembling/distributing reports and materials	'...making sure people got all the appropriate materials'; '...one of the most important things is just getting the information out to the group...'

Networking	'...making sure you have people that you can call if there are issues.'

Maintaining momentum and enthusiasm ('cheerleader')	'...the coaching and the keeping people motivated and kind of carrying the enthusiasm when it starts to lag a bit because the work can be a real grind.'

Ensuring group remains on task (and things are not missed)	'...getting your group together properly.'

Keeping group members informed	'...all the right people were informed.'

A key observation was that it is not necessarily the responsibility of one person to perform all the facilitation. Rather, it tends to be shared across a number of individuals on various levels. Participants described facilitation as a team effort. Some illustrative comments included: 'The idea that sort of runs through my mind is there are a number of people fulfilling these roles.' Further, this participant said, '...I see different people fitting into different pieces of this.' Another's idea was that '...the whole facilitation of this, it's really a team effort. It has, you know to, to make it successful you need a team to carry all this out.' However, when asked whether the emphasis is more on facilitation as a process rather than as an individual performing the facilitator role, one participant felt that there still is a need for '...some identified body [as] ...someone does have to coordinate the whole thing.'

Facilitators articulated additional comments on facilitation related to process. These areas were not evident in the case-audit documentation and were described as follows:

1. Making sure the correct people are involved in the project to ensure individuals with appropriate skill sets and content experts are included as well as to obtain buy in. One facilitator stated, '...we make sure the front-line staff and everyone's there but it's actually the managers and the hospitals and the administrators who actually have to be at the table.' This participant went on to say, 'It's great to get the staff online which we do fairly well for implementing change but we often forget the people who are actually making the decisions.'

2. Capacity building: As groups gain experience, the process and work '...then supports itself.' Facilitation can then '...become more of a project management kind of role and ensuring that things are followed up' and '...move more into the evaluative type of work and start up other groups that have no experience.'

3. Developing a close working relationship with the project lead or co-leads. This relationship was seen as a particularly important dynamic in driving the work forward. A good relationship between these individuals and good communication were seen as success factors and all participants agreed that 'without it, your project would fail.'

4. 'Having access to a venting office' as the adaptation process can take a lengthy period of time and cases may get frustrated with this. Participants identified that dealing with individuals' frustrations and need to talk about specific issues was a constant occurrence. One local facilitator described her office as '...the venting office. People that I'm working with come in and they rant and rave and vent and they feel better and then you know, we move on.'

We made specific enquiries regarding a few of the areas of facilitation for which little supporting evidence emerged from the case data. For example,

1. Taking on specific tasks did not emerge as part of the facilitator role. However, it was noted that facilitators '...end up actually doing the writing and doing the legwork and running around the building doing things rather than facilitating on a project-management level.' It's '...not just ensuring that other people are doing stuff on time...we're doing that plus we're actually doing it.'

2. Creating an open, supportive, and trusting environment conducive to change was another one of these areas but facilitators felt this was a part of what they do. This was associated with being supportive and inclusive and letting group members know that their input is valuable.

3. Facilitators also identified empowering group members as important and this involved not only empowering individuals but also entire groups.

Some other areas vocalized as important by facilitators but not prominent in the case-audit data were relationship building, teamwork, and helping to build in the structures and processes to support staff.

### Skills and knowledge for effective facilitation

Participants identified a range of expertise that they thought was essential for effective facilitation. Several key attributes repeatedly cited were:

1. Effective communication skills, ranging from maintaining regular close contact, ensuring the right individuals are informed and receive appropriate materials to a more complex awareness around communication on multiple levels. For example, one external facilitator described the need for '...a certain sensitivity and awareness around, particularly on multidisciplinary teams and sort of what the issues and challenges and context is around that requires a certain kind of communication sensitivity.'

2. Organizational skills, requiring military-style precision as described by one participant. Participants emphasized the importance of being organized and prepared, particularly for meetings. If meetings are not well run and organized, group members' time is seen as wasted and the work becomes delayed. As one local facilitator commented: '...you can be as cheerful and as kind and fantastic as you want but if you're not organized, you are dismissed. Like people will not pay any attention to you.'

3. Group dynamic and group leadership skills, involving making sure everyone is heard, assisting with shared decision making, and conflict resolution. This also included persuasiveness and negotiating skills.

4. 'Relational practice skills,' also described as relationship skills. According to one participant, this involved '...making people feel comfortable to express themselves, teambuilding, support, and encouragement.'

## Discussion

This facilitation study set out to explore and describe the facilitation activity occurring within a natural experiment where groups were beginning an evidence implementation. We followed three cases adapting and planning for implementation of guidelines designed to impact changes in nursing practice. The findings were revealing both conceptually and practically in terms of facilitating EBP through guidelines. First, it should be noted that in following these cases and not being prescriptive about process, we discovered the range and types of facilitation that groups themselves deemed necessary or at least helpful. They all utilized a combination of external and local facilitation. In two of the cases, external facilitators provided support and assistance primarily to local facilitators who in turn assisted cases. The other case engaged more direct external facilitation support and exhibited more of the facilitation activities performed by case members themselves. It was beyond the scope of the study, but more support may have been required because of limited resources or the planned implementation having a larger scope (*i.e*., national versus local).

The practical elements emerging from the literature, described as 46 discrete activities, were in large part found as occurring in the three cases. There were only three activities that no one, facilitators nor case members, engaged in. Two of these activities fell under the larger grouping of 'evaluating change.' This may be because cases are in the beginning stages of implementation and have not yet reached the point of evaluation. The third activity was 'creating an open, supportive, and trusting environment conducive to change.' Evidence for this activity may not have emerged in the documented data due to the nature of case-audit as a data collection method because certain activities are not always observable on paper. Facilitators when asked, however, felt this was part of their role. Further enquiry is needed to determine whether these elements actually represent facilitation in the practical sense and to further explicate the role of facilitation in evaluation of evidence implementation.

The case documents revealed an additional five distinct activities related to facilitation. The newly identified elements offer further insight into the role and process. A fundamental issue in all study cases was that members involved in the projects performed certain activities of facilitation in conjunction with or in addition to local and external facilitators. This is central to the notion of facilitation being considered a process and not something to be expected only of an individual as the 'facilitator.'

The focus group interview augmented and built upon the findings of the case audit, reinforcing the elements of facilitation identified, and offering new insights into the role and process. Although facilitators were appointed to their roles and each operationalized facilitation individually, all could relate to the activities and process identified. Participants also noted a range of requisite skills and knowledge is required for effective facilitation and provided practical examples from their experiences.

In line with Harvey *et al*.'s [3] findings, facilitators performed a vast array of activities ranging from practical, task-oriented assistance to providing holistic and enabling support. The results should be interpreted with caution because the facilitation activity was considered performed even if engaged in by one facilitator in one case. However, the activities were generally performed by facilitators across cases. The activities engaged in by local and external facilitators across all cases could be considered key elements of the process (Table 3). When asked, facilitators themselves spontaneously identified many of these same elements as important. Although these findings have limited generalizability beyond these cases, they support the understanding of facilitation in the literature and add strength to descriptions of the concept.

There appeared to be some overlap between the local and external facilitator roles. For example, a large part of both roles involved overcoming others' resistance to change, providing resources/tools, and problem solving. However, the roles differed in that local facilitators took on the majority of the administrative and project-specific assistance, including data management, whereas external facilitators provided more general, ongoing support and reassurance. Interestingly, local facilitators recognized the external facilitators as 'expert' in relation to guideline adaptation and EBP processes and as such, the external facilitators were seen as bringing external credibility to the projects.

It is important to note that although cases performed certain facilitative activities, facilitators considered capacity-building to be an important element of their role. Facilitators were doing some but not all of the legwork while at the same time helping the cases to develop the skills and confidence to facilitate guideline adaptation. To a large extent, facilitators were helping the group develop the capacity to do it themselves as opposed to doing it for them. In this way, the role was not entirely prescriptive or rather largely nondirective but somewhere in the middle.

Administrative and project management emerged as important aspects of facilitation. It was noted in the case data that facilitators organized, scheduled, and often led meetings and were influential in ensuring the case remained on task. Other authors identify the potential overlap between facilitation and project management [5]. This has been previously explained in a different sense whereby facilitators are described as project leads [12]. The context in our study was different. In addition to a dedicated local facilitator, each case had an appointed project lead or co-leads selected for the position(s) due to their experience with guideline development and/or content area expertise. Participants in the focus group interview recognized that case members, particularly project leads, do not have the time to carry out the administrative tasks of organizing meetings and the follow up associated with keeping cases on track. Therefore, facilitators across cases took on these tasks.

We also found that facilitators used approaches which could be considered elements of other implementation interventions. For example, facilitators provided education to case members to increase their interpretation of the research evidence in order to adapt the knowledge to their environment and also assisted them with obtaining skills training in guideline adaptation and literature searching and appraisal. As well, facilitators supported and in some cases, organized and performed audit and feedback mechanisms to provide case members with information regarding their clinical practice performance. Facilitation process also involved components of a linking agent role namely, boundary-spanning and liaising between case members and other individuals at multiple levels, involved or uninvolved in the project, to obtain necessary resources. Similar to Stetler *et al*.'s [5] findings, facilitation could be deemed a mediating process or intervention because it involves organizing and enabling actualization of other change strategies.

These findings have important implications for those planning an evidence implementation. It is important to consider the different roles of all individuals involved. Recognition of the range of activities associated with facilitation and corresponding requisite knowledge and skills will be useful for selecting individuals to fulfill this type of role and in developing facilitator training programs. Despite there being some overlap between local and external facilitator roles, there are particular differences we have noted that should be considered in filling these roles and ensuring the appropriate people are involved in adaptation and implementation. Depending on the additional resources required, obtaining the assistance of an external facilitator with professional development and/or guideline implementation expertise may be useful in bringing external credibility to a project.

Our findings are consistent with the definition of facilitation put forth by Stetler *et al. *[5]: 'a deliberate and valued process of interactive problem solving and support that occurs in the context of a recognized need for improvement and a supportive interpersonal relationship' [5]. Across cases, both local and external facilitators engaged in activities related to problem solving and addressing specific issues and provided ongoing support and reassurance to case members.

As well, facilitators perceived effective communication and relationship building, particularly with project leads, to be key elements of the role. In a practice environment, the lead(s) might be the unit quality council or nurse manager who leads an implementation. The audit data also reflected the importance of communication. Both local and external facilitators fulfilled most of the activities under 'effective communication' according to the audit tool and provided regular communication and kept case members informed across all three cases. As described in the literature, enhancing relationships and fostering relationship building are components of facilitation [13,14] along with themes of strong interpersonal and communication skills [3,5,6].

A key observation in this study is that facilitative activities tend to be shared across a number of individuals. This validates what was discovered in the literature review that facilitation is now being viewed as both an individual role as well as a process involving individuals and groups [4]. Facilitators perceived facilitation as a team effort. This is important to consider in planning implementation work in relation to evaluating both the strengths and weaknesses of group members and ties into ensuring the correct individuals are involved. Participants identified that there is a need for a recognized individual, such as a facilitator, to coordinate the group. However, groups may bring their own assets and possess certain facilitative skills which could be capitalized on.

For example, Kitson *et al. *[1] proposed that facilitators play a key role in helping individuals understand what they need to change and how to make these changes to incorporate evidence into practice. Our results indicated that case members themselves were able to identify a need for practice change recognized as a priority by staff. This may be because case members had already identified an area for change prior to volunteering to participate in the guideline adaptation process. However, it begs the question of whether there are certain activities of facilitation that are more applicable if facilitated by case members themselves provided they have the internal capacity and resources. As cases were able to identify a problem area in their clinical practice, facilitators could spend more time focusing on assisting cases with 'how' to change practice as opposed to finding 'what' needs to be changed. Group members in all cases also identified leaders for their respective projects. This may be another area of facilitation more appropriate for group members to address themselves. Cases may be more cognizant of who amongst their colleagues would make a qualified leader as opposed to a facilitator being brought in to assist a group with a particular project.

### Study limitations

Study findings should be interpreted in consideration of some limitations. First, facilitators were appointed or hired for the role, and this may have had an effect on their responses. It may have been beneficial to also seek the case members' perceptions on facilitation. Secondly, a potential source of bias was encountered in that one author (EJD) extracted data from the case manuals and categorized the evidence. Finally, we observed the facilitation taking place in three in-depth cases in the early stages of evidence implementation activity with a focus on adapting guidelines for use by nurses. Facilitation process may be applied differently in regards to changing the practice of different professional groups, particularly in regards to the dynamics of their practice (*i.e*., physicians may have somewhat more autonomy and independence in some areas of their practice whereas nurses largely work in teams). As well, although the cases focused on different guideline topics areas, all focused on one broader area of care (cancer) which may limit generalizability because there are distinct health delivery and system features in this area. Context is an important factor that relates to the facilitation approach taken; thus, the facilitation occurring in these cases may or may not be generalizable to other disciplines, points in the implementation process, or in other situations or settings.

Using multiple methods of data collection, we attempted to lessen bias through triangulation of different data sources. Data collected included group processes (*i.e*., meetings, communications as well as self reports, field notes) and a focus group interview with facilitators. To lessen the effect of potential bias in having one author interpret and categorize the evidence, the data manager checked the evidence supporting the categories and confirmed or challenged these interpretations. A detailed record of this discussion was kept. As well, facilitators' perceptions were sought as to whether the categories and activities in the audit fit or were misrepresented based on their own experiences. The context of the cases is described to inform potential transferability of the findings.

## Conclusions

This study highlights some of the key, practical and conceptual elements of facilitation as a role and process in early implementation of EBP in nursing. The distinct facilitation activities identified and facilitator perceptions offer a comprehensive description of the concept in real situations. Practical aspects of facilitation expand what is known and further validate what has been previously noted in the literature. With a better understanding of what facilitation entails, future research should concentrate on evaluating the effectiveness of facilitation interventions, both local and external, in influencing changes in nursing practice. This will not be easy because facilitation is a multifaceted and complex role and process. Therefore, it is important that the research methods and facilitation interventions employed in future research be described in detail. This information could then be used to develop and expand the contribution of facilitation as a means of bridging the gap between research and practice.

## Competing interests

The authors declare that they have no competing interests.

## Authors' contributions

EJD and MBH conceived of the study and participated in the design. EJD was primarily responsible for study coordination, data collection, analysis and interpretation, and initial draft of the manuscript and subsequent revisions. MBH provided initial and ongoing refinements to the manuscript as well as critique of the data analysis and interpretation and synopsis of findings. IDG and CB provided feedback on study design and contributed to conceptualization of data analysis. All authors critiqued the data analysis and interpretation and synopsis of findings. All authors provided editorial contributions and read and approved the final manuscript.

## Supplementary Material

Additional file 1 **Facilitation case-audit tool**. Excel Spreadsheet (XLS) displaying the blank audit tool which was used to analyze documentary data from the individual case manuals. Click here for file

Additional file 2 **Facilitation across cases (local, external, case members)**. Excel Spreadsheet (XLS) displaying the types of facilitation activities identified and who performed them across cases. Click here for file
